# Enhancement of Wheat Seed Germination, Seedling Growth and Nutritional Properties of Wheat Plantlet Juice by Plasma Activated Water

**DOI:** 10.1007/s00344-022-10677-3

**Published:** 2022-05-31

**Authors:** Junhong Wang, Jun-Hu Cheng, Da-Wen Sun

**Affiliations:** 1 School of Food Science and Engineering, South China University of Technology, Guangzhou, 510641 China; 2 Academy of Contemporary Food Engineering, South China University of Technology, Guangzhou Higher Education Mega Center, Guangzhou, 510006 China; 3 Engineering and Technological Research Centre of Guangdong Province On Intelligent Sensing and Process Control of Cold Chain Foods, & Guangdong Province Engineering Laboratory for Intelligent Cold Chain Logistics Equipment for Agricultural Products, Guangzhou Higher Education Mega Centre, Guangzhou, 510006 China; 4 Food Refrigeration and Computerized Food Technology (FRCFT), Agriculture and Food Science Centre, University College Dublin, National University of Ireland, Belfield, Dublin 4, Ireland

**Keywords:** Nutritional properties, Plasma-activated water, Seed germination, Seedling growth, Wheat plantlet juice

## Abstract

Previous studies have shown the great potential of using plasma-activated water (PAW) on improving agriculture seed germination, however, information on the influence of PAW on crop plantlet juice remains scanty. In this research, the effect of PAW generated by atmosphere pressure Ar–O_2_ plasma jet for 1–5 min on wheat seed germination, seedling growth and nutritional properties of wheat plantlet juice was investigated. Results revealed that all PAWs could enhance wheat seed germination and seedling growth in 7 days by improving the germination rate, germination index, fresh weight, dry weight and vigour index, and especially that PAW activated for 3 min (PAW-3) showed the best overall performance. In addition, the application of PAWs enhanced the nutritional properties of wheat plantlet juice from those grown for 14 days by improving total soluble solids, protein content, photosynthetic pigments, total phenolic content, antioxidant activity, enzyme activity, free amino acids and minerals content, and the best enhancement was also observed in PAW-3. It was concluded that PAWs would be an effective technique to enhance the growth and nutritional properties of crop sprouts, which could be served as functional foods in many forms.

**Supplementary Information:**

The online version contains supplementary material available at 10.1007/s00344-022-10677-3.

## Introduction

With the rapid increase of the world population and public awareness, demands for quantity and quality of food have continuously been elevated. However, nearly 690 million people in the world still lack adequate food supply, and this number will exceed 840 million by 2030 even if the potential impact of the COVID-19 outbreak is set aside (FAO et al. 2020). Therefore, it is important to develop more efficient and sustainable crop production methods to address the basic food need of people. It is well known that wheat (*Triticum aestivum* L.) is one of the most important crop plants supplying staple food throughout the world. The nutritional value of wheat comes from its high content of carbohydrates, proteins and dietary fibres, as well as considerable proportions of vitamins, minerals and antioxidants such as carotenoids, phenolics and phytosterols (Abdel-Aal and Rabalski 2008). After germination, wheat sprouts are a homology of medicine and food, which are rich in bioactive compounds and could be consumed as raw juice, tablets and capsules (Akbas et al. 2017), and wheat plantlet juice is thus increasingly being studied (Ahmed et al. 2020; Qamar et al. 2019; Sharma et al. 2020). Therefore, improving wheat seed germination and growth is of great importance to the industry.

Traditional methods in improving wheat seed germination are mainly based on chemical treatments, which can cause environmental hazards; therefore, novel physical treatment techniques have been investigated for their potential enhancement effects on wheat seed germination and seedling growth, which includes treatments by ultrasound (Guimarães et al. 2020), pulsed electric field (Ahmed et al. 2020), irradiation (AlSalhi et al. 2019), magnetic field (Balakhnina et al. 2015) and cold plasma (Kučerová et al. 2019; Lotfy et al. 2019; Roy et al. 2018). Amongst these techniques, cold plasma is the most impressive and the most studied in the latest decades (Chen et al. 2020; Chizoba Ekezie et al. 2017; Jiang et al. 2020; Pan et al. 2019a).

Cold plasma generated by electrical discharges is known for its active species such as UV light, radicals, reactive oxygen species (ROS) and reactive nitrogen species (RNS), and environment-friendly properties, which lays the foundation for its applications in agriculture and food processing (Scholtz et al. 2019; Song et al. 2020; Ali et al. 2022; Esua et al. 2021; Han et al. 2019a; Pan et al. 2021; Wu et al. 2022; Zhu et al. 2022). There are two different ways of applying cold plasma for seed treatments in agriculture: direct treatments, which are frequently used in previous research and have shown positive impacts on crop growth (de Groot et al. 2018; Ling et al. 2014; Velichko et al. 2019; Xu et al. 2019), and indirect treatments by using plasma-activated water (PAW) (Esua et al. 2021; Esua at al. 2022a; Esua et al. 2022b).

Compared with the direct treatment, PAW is easier to realize the uniform treatment of crop seeds and the storage and transport of plasma active species. PAW is typically generated by applying cold plasma on the surface of water or underneath the water with various plasma sources, and the composition of PAW depends on the plasma-liquid interactions, which usually leads to changes in pH, electrical conductivity (EC) and especially the formation of reactive oxygen and nitrogen species (RONS) (Ndiffo Yemeli et al. 2020; Ali et al. 2021a; Esua et al. 2022c; Pan et al. 2020). Several studies have identified that the long-life species (H_2_O_2_, NO_2_^−^ and NO_3_^−^) are the source of activity of PAW. Although short-life species have higher physiological activity, they disappear soon after being generated (Asim et al. 2020; Bradu et al. 2020; Zhou et al. 2020).

Previous studies have indicated the positive effects of PAW on seed germination and seedling growth. Adhikari et al. (2019) investigated the effect of PAW on tomato seedlings by applying distilled water exposed to cold atmospheric-air jet plasma for 15, 30 and 60 min and found that PAW irrigation could upregulate seedling growth, endogenous RONS, defence hormones and expression of pathogenesis-related genes, which depended on the RONS concentration in PAW. Gierczik et al. (2020) clarified that PAW could improve the tolerance of barley against combined low temperature and hypoxia stresses during germination for the effect on the antioxidant system by RONS. Similarly, Zhang et al. (2017) compared the effect of tap water, PAW and commercial fertilizer on lentil germination and stem growth and showed that PAW increased the germination rate to 80% as compared with 30% using tap water, and the stem lengths were better enhanced than using commercial fertilizer, which was strongly related to H_2_O_2_ and NO_3_^−^ in PAW. In addition, Kučerová et al. (2019) studied the effects of PAW prepared by transient spark discharge on wheat in vitro and in vivo and revealed that PAW might effectively stimulate the growth of wheat seedlings and positively affect their metabolism, which was correlated with the concentration of RONS in the PAW. However, the research about the effect of PAW on the nutritional properties of crop plantlets or their juice remains scanty at present.

Therefore, the objective of the current study was to investigate the influence of PAW generated by Ar–O_2_ plasma for 1–5 min on wheat seed germination, seedling growth and nutritional properties of wheat plantlet juice. It is hoped that the current study should provide an effective method to accelerate the growth and nutrition enrichment of crop plants.

## Materials and Methods

### Materials and Chemicals

Fresh wheat (*Triticum aestivum* L.) seeds (Jimai 23) used in the current study were supplied by Shandong Luyan Agricultural Co., Ltd. (Shandong, China). Uniform wheat kernels based on shape, size and colour were selected and debris and damaged seeds were removed from the experimental samples. Ascorbic acid (ASA), 2,6-dichloroindophenol (DIP), 2,2’-azobis-amidinopropane (ABAP), Folin-Ciocalteu reagent and gallic acid (GA) were purchased from Sangon Biotech Co., Ltd. (Shanghai, China). 2,2-Diphenyl-1-picrylhydrazyl (DPPH), sodium carbonate (Na_2_CO_3_), acetone, hydrochloric acid (HCl), metaphosphoric acid and potassium nitrate (KNO_3_) were obtained from Aladdin Reagent Co., Ltd. (Shanghai, China). Enzyme activity assay kits were acquired from Shanghai Yuanye Bio-Technology Co., Ltd. (Shanghai, China). The hydrogen peroxide assay kit was bought from Nanjing Jiancheng Bioengineering Institute (Nanjing, China). Distilled water was procured from Watsons Co., Ltd. (Guangzhou, China).

### Experimental Setup for PAW Production

**Fig. 1:**
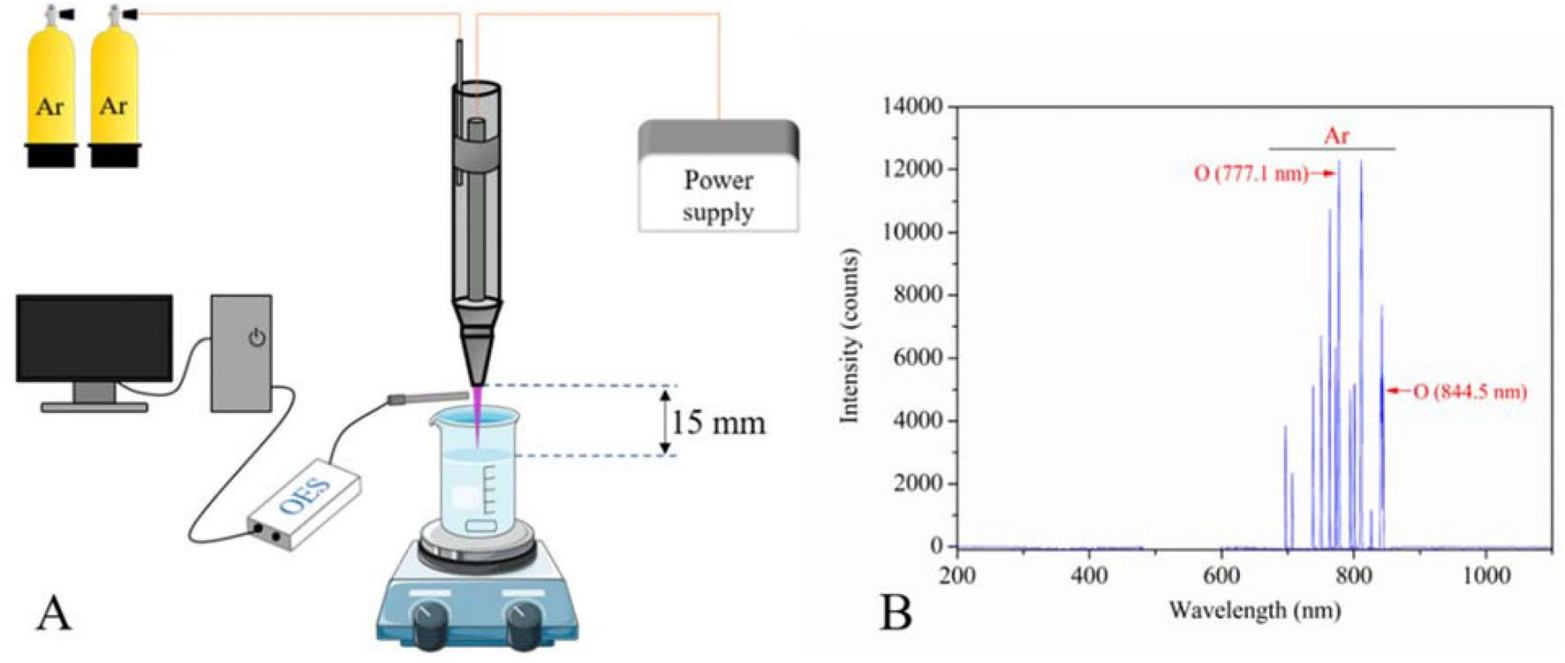
**A** Experimental setup of the plasma jet system and **B** optical emission spectrum of Ar–O_2_

The plasma-generating device employed in the current study was an atmospheric pressure plasma jet (UPL-310B, Uniplasma Co., Ltd, Shenzhen, China), which has been previously described in detail by our lab members (Chizoba Ekezie et al. 2018; Han et al. 2019b; Chizoba Ekezie et al. 2019a; Chizoba Ekezie et al. 2019b; Pan et al. 2019b). The scheme of the plasma jet and the arrangement for the treatment of distilled water are shown in Fig. 1A. The plasma was powered by an alternating current single-phase power source at 220 V with an output voltage of 7 kV and the working power of the device was fixed to 600 W. Mixed gas (98% Ar and 2% O_2_) was used as the feed gas with a fixed gas flow rate of 40 L/min. For the production of PAWs, 75 mL of distilled water were poured into a 100 mL glass beaker (4.6 × 7.2 cm, diameter × height) and exposed to the plasma jet for a period of 1, 2, 3, 4 and 5 min, respectively. The length of the plasma plume was about 14 mm and the distance between the nozzle of the plasma jet and the water level in the beaker was set to 15 mm to increase gas–liquid interactions. The beaker was shaken constantly by a magnetic stirring apparatus (ZGCJ-3A, Zigui Co., Ltd., Shanghai, China) during plasma jet treatment to ensure a homogeneous distribution of active species. The temperature of the water in the beaker was monitored by a digital thermometer (ZG-8029S, Chigo Co., Ltd., Ningbo, China) before and after the treatment. After treatment, water samples treated by plasma for 1–5 min were designated as PAW-1, PAW-2, PAW-3, PAW-4 and PAW-5, respectively, and these PAWs were used for testing or irrigating wheat seeds within 30 min to prevent the changes of PAW and ensure repeatability. Untreated distilled water was used as control and designated as DW.

The plasma source was analysed by a computer-controlled optical emission spectrometer (OES) (HR2000+, Ocean Optics, Inc., FL, USA) equipped with a fibre optic (0.22 µm). The spectra were obtained in the range from 200 to 1100 nm. The axial and radial distances between the middle of the nozzle and the detector were both approximately 5 mm. Identification of the peaks was carried out by comparing experimental results with the National Institute of Standards and Technology (NIST) Atomic Spectra Database.

### Characterization of PAWs

The characterization of PAW was carried out by measuring pH, electric conductivity (EC), NO_2_^−^, NO_3_^−^ and H_2_O_2_ content. The pH and EC values were measured by a pH meter (SP-2300, Suntex Instrument Co., Ltd., Taiwan, China) and a conductivity meter (DDS-11A, Leici Chuangyi Instrument & Meter Co., Ltd., Shanghai, China), respectively. The contents of NO_2_^−^ and NO_3_^−^ were measured by methods reported previously (Shen et al. 2016). Nitrite concentration in PAWs was quantified with Griess reagent consisting of an equal volume of 10 g/L sulfanilic acid and 1.0 g/L naphthol. Briefly, 10 mL PAW was mixed with 0.5 mL Griess reagent and incubated at room temperature for 20 min. Then the absorbance of the mixture was determined by a spectrophotometer (UV-1800, Shimadzu Co., Kyoto, Japan) at 540 nm and nitrite solution (0.1–1.0 mg/L) was used as the standard for calculation. As for NO_3_^−^, 20 mL PAW was added with 1.0 mL of 1% sulfamic acid and diluted to 50 mL with distilled water. The absorbances at 220 nm (A220) and 275 nm (A275) of the mixture were determined, and nitrate concentration was calculated by the difference between A220 and A275 according to the standard curve of nitrate solution (0–8.0 mg/L). The H_2_O_2_ content was determined using a hydrogen peroxide assay kit (A064-1-1, Nanjing Jiancheng Bioengineering Institute, Nanjing, China) based on the photometrical determination at 405 nm after the reaction of H_2_O_2_ with molybdic acid (Xiang et al. 2018). The results were calculated according to the standard curve of H_2_O_2_ solution (0–16.3 mmol/L). 

### Germination of seeds

 To evaluate the effect of PAWs on wheat seed germination and seedling growth, germination tests for 7 days were performed. In total, there were 6 samples with 3 replications per sample and each replication contained 50 wheat seeds. Seeds were germinated in a germination box containing one layer of filter paper and watered with the same volume of DW, PAW-1, PAW-2, PAW-3, PAW-4 and PAW-5, respectively, to keep the moisture needed for seed germination. The germination boxes were placed in an illumination incubator with a temperature of 20 ± 1 ℃ and relative humidity of 70 ± 5% during the whole germination test. After 3 days, 2000 lx light for 12 h was added every day. The number of germinated seeds was observed and recorded daily. After germination for 7 days, 15 seedlings of each box were randomly selected for fresh and dry weight determination. Germination rate, germination index and vigour index were calculated according to the following equations:

1 $${\text{Germination}}\,{\text{rate}}~ = ~\frac{{{\text{seeds}}\,{\text{germinated}}}}{{{\text{total}}\,{\text{seeds}}}}\, \times 100\%$$

2 $${\text{Germination}}\,{\text{index}}~ = ~\sum \frac{{{\text{the}}\,{\text{number}}\,{\text{of}}\,{\text{seeds}}\,{\text{germinated}}\,{\text{in}}\,{\text{one}}\,{\text{day}}}}{{{\text{the}}\,{\text{number}}\,{\text{of}}\,{\text{days}}}}$$

3 $${\text{Vigour}}\,{\text{index}}\,{\text{A}}~ = ~{\text{Germination}}\,{\text{index}}~ \times ~{\text{Mean}}\,{\text{fresh}}\,{\text{weight}}$$

4 $${\text{Vigour}}\,{\text{index}}\,{\text{B}}~ = ~{\text{Germination}}\,{\text{index}}~ \times ~{\text{Mean}}\,{\text{dry}}\,{\text{weight}}$$

### Juice Preparation of Wheat Plantlets

To evaluate the effect of PAWs on the quality and nutritional characteristics of wheat plantlet juice, pot experiments for 14 days were conducted. For the growth of wheat plantlets, 24 pots were divided into 6 groups, each containing 180 g of vermiculite and 200 mL of DW, PAW-1, PAW-2, PAW-3, PAW-4 and PAW-5, respectively. In each group, 25 g of seeds were used, which were divided on average into 4 replications and the seeds were sowed uniformly 2 cm underneath the surface of the vermiculite. All the pots were placed inside the laboratory where sufficient light and airflow were present. During the growth period, the weight of each pot was recorded every day in order to add an appropriate amount of DW or PAWs in time. Wheat plantlets watered with DW or PAWs were designated as DW wheat plantlets, PAW-1 wheat plantlets, PAW-2 wheat plantlets and so on, and were cut with scissors after 14 days, 1 cm above the surface. Wheat plantlet juice was extracted with the cold pressing method by using a specialized manual juice extractor (MM-XP01, Linyi Maimiao Trading Co., Ltd., Linyi, Shandong), which could prevent oxidation and heat. After extraction, wheat plantlet juice was centrifuged immediately at 10,000 rpm for 5 min using a centrifuge (JW-3024HR, Anhui Jiaven Equipment Industry Co., Ltd., Hefei, China), which was then divided into 2 mL centrifuge tubes and stored at -20 ℃ for further determination. All the tests were accomplished within one week.

### Physical and Chemical Analysis of Wheat Plantlet Juice

#### pH, Total Soluble Solids and Colour

The pH value of wheat plantlet juice was determined by the pH meter. The total soluble solids (TSS) were determined by dropping 300 μL of wheat plantlet juice into a digital refractometer (Atago Co., Ltd., Tokyo, Japan) at room temperature of 25 °C and the results were expressed as °Brix. The colour assessment was conducted at room temperature of 25 °C using a CR-400 colourimeter (Konica Minolta Inc., Osaka, Japan), and the hunter L^*^ (lightness), a^*^ (redness or greenness), and b^*^ (yellowness or blueness) values of wheat plantlet juice were measured and the total colour difference (△E^*^) was calculated using the equation below:

5 $$\Delta E^{*} ~ = ~\sqrt {(L_{{{\text{control}}}}^{*} ~ - ~L^{*} )^{2} ~ + ~(a_{{{\text{control}}}}^{*} ~ - ~a^{*} )^{2} ~ + ~(b_{{{\text{control}}}}^{*} ~ - ~b^{*} )^{2} }$$

#### Vitamin C Contents

The vitamin C content of wheat plantlet juice was determined using the 2,6-dichloroindophenol (DIP) titrimetric method as reported previously (Wang et al. 2017) with some modification. Briefly, 1.0 mL wheat plantlet juice and 9.0 mL chilled 2% (w/v) metaphosphoric acid buffer were added to a 50 mL conical flask for titration. Ascorbic acid (ASA) was used as the standard for calculation, and the content of vitamin C was expressed as milligram per 100 g of wheat plantlets in fresh weight (mg/100 g FW).

#### Soluble Protein Contents

Soluble protein was assayed according to the Bradford method (Ling et al. 2015). Briefly, 20 μL of appropriately diluted wheat plantlet juice was mixed well with 1.0 mL coomassie brilliant blue G-250 solution and the absorbance of the mixture was determined after 2 min using the spectrophotometer at 595 nm. Bovine serum albumin (BSA) was used as the standard for calculation and the results were expressed as grams per 100 g of wheat plantlets in fresh weight (g/100 g FW).

#### Pigments Contents

The values of chlorophyll a (${\text{C}}_{\text{a}}$), chlorophyll b (${\text{C}}_{\text{b}}$) and carotenoids (${\text{C}}_{\text{x + c}}$) were measured by the spectrophotometer at 646.8, 663.2 and 470 nm (Lichtenthaler & Buschmann, 2001). Concretely, 1.0 mL of properly diluted wheat plantlet juice was mixed with 4.0 mL of acetone and 50 mg of CaCO_3_. After standing for 5 min, the mixture was centrifuged at 10,000 rpm for 5 min and the absorbances at 646.8, 663.2 and 470 nm for the supernatant were measured, respectively. The amounts of pigments present in the juice samples were calculated according to the formula below:

6 $${\text{C}}_{\text{a}}{ = 12.25}{\text{A}}_{663.2}- \text{ 2.79}{\text{A}}_{646.8}$$

7 $${\text{C}}_{\text{b}}{ = 21.50}{\text{A}}_{646.8}- \text{ 5.10}{\text{A}}_{663.2}$$

8 $${\text{C}}_{\text{x + c}}{ = }\frac{{1000}{\text{A}}_{470} -\text{ 1.82}{\text{C}}_{\text{a}}- \text{85.02}{\text{C}}_{\text{b}}}{198}$$

The results were expressed as μg per 100 g of wheat plantlets in fresh weight (μg/100 g FW).

#### Total Phenolic Contents

The total phenolic content (TPC) of wheat plantlet juice was quantified by the Folin-Ciocalteu reagent method as modified by Xiang et al. (2017). Gallic acid (GA) was used as the standard for calculation. The results were calculated according to the standard curve of gallic acid concentrations (0–600 μg/mL) and expressed as milligram gallic acid equivalent (GAE) per 100 g of wheat plantlets in fresh weight (mg GAE/100 g FW).

#### Antioxidant Activities

Antioxidant activities of wheat plantlet juice were evaluated using the DPPH free radical scavenging assay (Ali et al. 2019) and oxygen radical absorbance capacity (ORAC) assay (Xiang et al. 2017).

For the DPPH assay, 100 μL of diluted wheat plantlet juice was mixed with 900 μL of 150 μmol/L DPPH and then incubated at room temperature of 25 °C for 30 min in the dark. Distilled water and vitamin C were used as the blank control and Trolox standard, respectively. The absorbance at 519.5 nm was measured and used to calculate the percentage of quenched DPPH. The results were calculated according to the standard curve of vitamin C concentrations (0–400 μg/mL).

For ORAC assay, 20 μL of appropriately diluted wheat plantlet juice was added with 200 μL of 0.96 μM fluorescein and incubated at 37 ℃ for 20 min with intermittent shaking. After adding 20 μL of 119.4 mM ABAP reagent, the fluorescence intensity was monitored every 4.5 min for 35 cycles at excitation of 485 nm and emission of 535 nm by a Tecan Infinite™ M200 Multimode Microplate Reader (Tecan Inc., Männedorf, Switzerland).

The results of antioxidant activities were expressed as μmol Trolox equivalent (TE) per gram of wheat plantlets in fresh weight (μmol TE/g FW).

#### Enzyme Activities

The activities of superoxide dismutase (SOD), peroxidase (POD) and polyphenol oxidase (PPO) in wheat plantlet juice were analysed by assay kits R22261, R30312 and R30314 (Shanghai Yuanye Bio-Technology Co., Ltd., Shanghai, China), respectively.

For the SOD assay, the nitroblue tetrazolium (NBT) method was used. The absorbance was determined at 560 nm after 20 min light reaction and the light-avoiding tube was used as control. The enzyme activity unit was defined as inhibiting 50% of NBT reduction.

For the POD assay, the guaiacol method was used. The absorbance was recorded continuously at 470 nm for 3 min and the sample boiled for 5 min was used as the control. Enzyme activity unit was defined as the amount of enzyme required to change absorbance by 0.01 per minute.

For the PPO assay, the catechol method was used. The absorbance was recorded continuously at 420 nm for 1 min and the boiled samples were used for the blank control. Similarly, the enzyme activity unit was defined as the amount of enzyme required to change absorbance by 0.01 per minute.

The measured values were expressed as enzyme activity unit per gram of wheat plantlets in fresh weight (U/g FW).

#### Free Amino Acids

The free amino acids of wheat plantlet juice were measured by an L-8900 amino acid analyzer (Hitachi Inc., Tokyo, Japan). Briefly, 4.0 mL of 15% (w/v) sulfosalicylic acid was added to 1.0 mL of wheat plantlet juice and mixed well to precipitate protein. Then, the mixture was centrifuged at 10,000 rpm for 5 min and the supernatant was collected and diluted properly for detection. Free amino acids were separated by liquid chromatography with a column (855–4507, Hitachi Inc., Tokyo, Japan) and detected through ninhydrin reaction at 570 nm and 440 nm. External standards (Sigma-Aldrich Trading Co., Ltd., Shanghai, China) were used for concentration calculation. All values were described in milligram per 100 g wheat plantlets in fresh weight (mg/100 g FW).

#### Mineral Elements

Mineral contents of wheat plantlet juice were estimated using an inductively coupled plasma-optical emission spectrometer (iCAP 7200, Thermo Scientific™, MA, USA). Briefly, 1.0 mL of juice sample was poured into a Teflon vessel with the addition of 4.0 mL of 65% HNO_3_ and 1.0 mL of 30% H_2_O_2_ for digestion on a microwave work station (Mars 6, CEM Co., Matthews, NC, USA). After that, digested samples were diluted with ultrapure water to 50 mL for measurement. For each mineral compound, the standards were prepared within the range of the concentration of mineral elements contained in the sample. The results were described in micrograms per gram of wheat plantlets in fresh weight (μg/g FW).

### Statistical Analysis

Data were presented as mean ± standard deviation (SD) for at least three independent experiments. Statistical analysis was performed using SPSS software for Windows (V 22.0, IBM Co., New York, USA). Significance tests of data were performed by applying one-way analysis of variance (ANOVA) and Duncan’s multiple comparison post-test with a confidence level of 95%. A *p* value less than 0.05 was regarded as statistically significant. Correlation tests between the PAW characteristics and the observed effects in wheat were performed using Pearson’s correlation. Significant differences were represented by *p < 0.05 and **p < 0.01.

## Results

### Emission Characteristics of Plasma Discharge

To examine the emission spectrum generated by the atmospheric pressure plasma jet, OES was used. As shown in Fig. 1B, the optical emission spectrum of the discharge for the Ar–O_2_ plasma jet was mainly comprised of the emission lines of argon and its excited states as Ar was the main constituent of the feed gas. In the meantime, the emission lines of atomic oxygen at 777.1 and 844.5 nm were also observed, suggesting that argon and oxygen reactive species were responsible for the activation of the treated water.

### Physicochemical Properties of PAWs

**Fig. 2:**
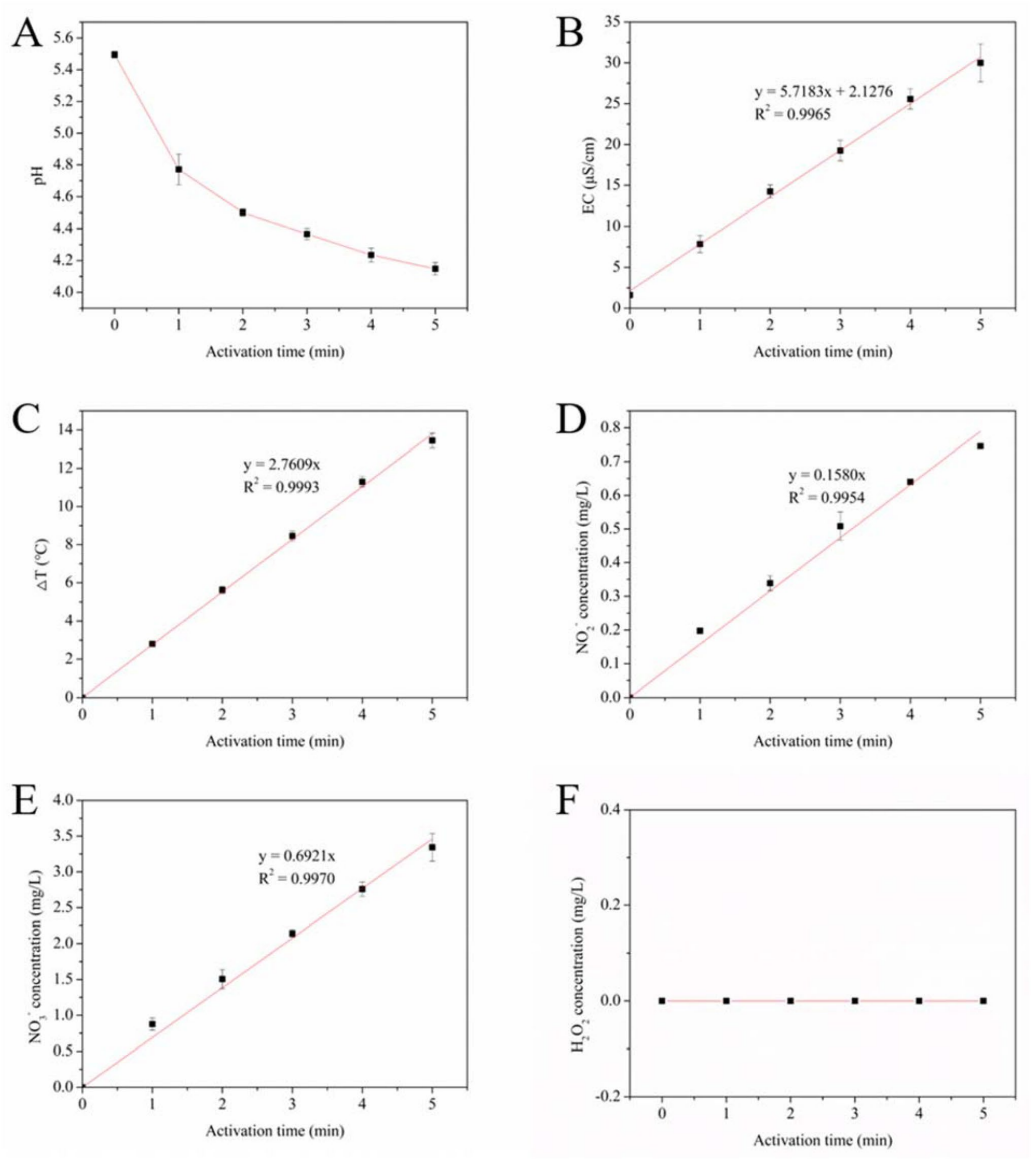
**A** pH, **B** EC, **C** temperature variation, **D** NO_2_^−^ concentration, **E** NO_3_.^−^ concentration and **F** H_2_O_2_ concentration of PAWs as affected by plasma activation times

To clarify the properties of PAWs, the changes in the pH, EC, temperature, and NO_2_^−^, NO_3_^−^ and H_2_O_2_ concentrations of PAWs after plasma treatment were monitored and presented in Fig. 2. In general, with an increase in plasma application time (activation time), the pH of the PAWs decreased, whilst the EC, temperature, and NO_2_^−^ and NO_3_^−^ concentrations increased and the H_2_O_2_ concentration remained zero. For the pH, with applying plasma, the pH of PAWs started to drop and after activation for 5 min, the value decreased to 4.15 ± 0.04 from an initial value of 5.50 ± 0.02. For EC, the change tendency was totally opposite to that of the pH, and the highest EC value of 30.00 ± 2.32 μS/cm occurred in PAW-5 as compared with an initial value of 1.61 ± 0.09 μS/cm for DW. Similarly, the temperature of PAWs increased until the plasma treatment was finished, and the highest temperature rise appeared in PAW-5 with 13.45 ± 0.39 ℃, indicating the nature of cold plasma treatment. For NO_2_^−^ and NO_3_^−^ concentrations, their changing patterns were the same as that of temperature or EC, and the values increased linearly from an initial zero to 0.75 ± 0.00 and 3.34 ± 0.19 mg/L for PAW-5, respectively. These results suggested that the composition of PAWs was strongly dependent on activation time.

### Germination and Growth Parameters

**Fig. 3:**
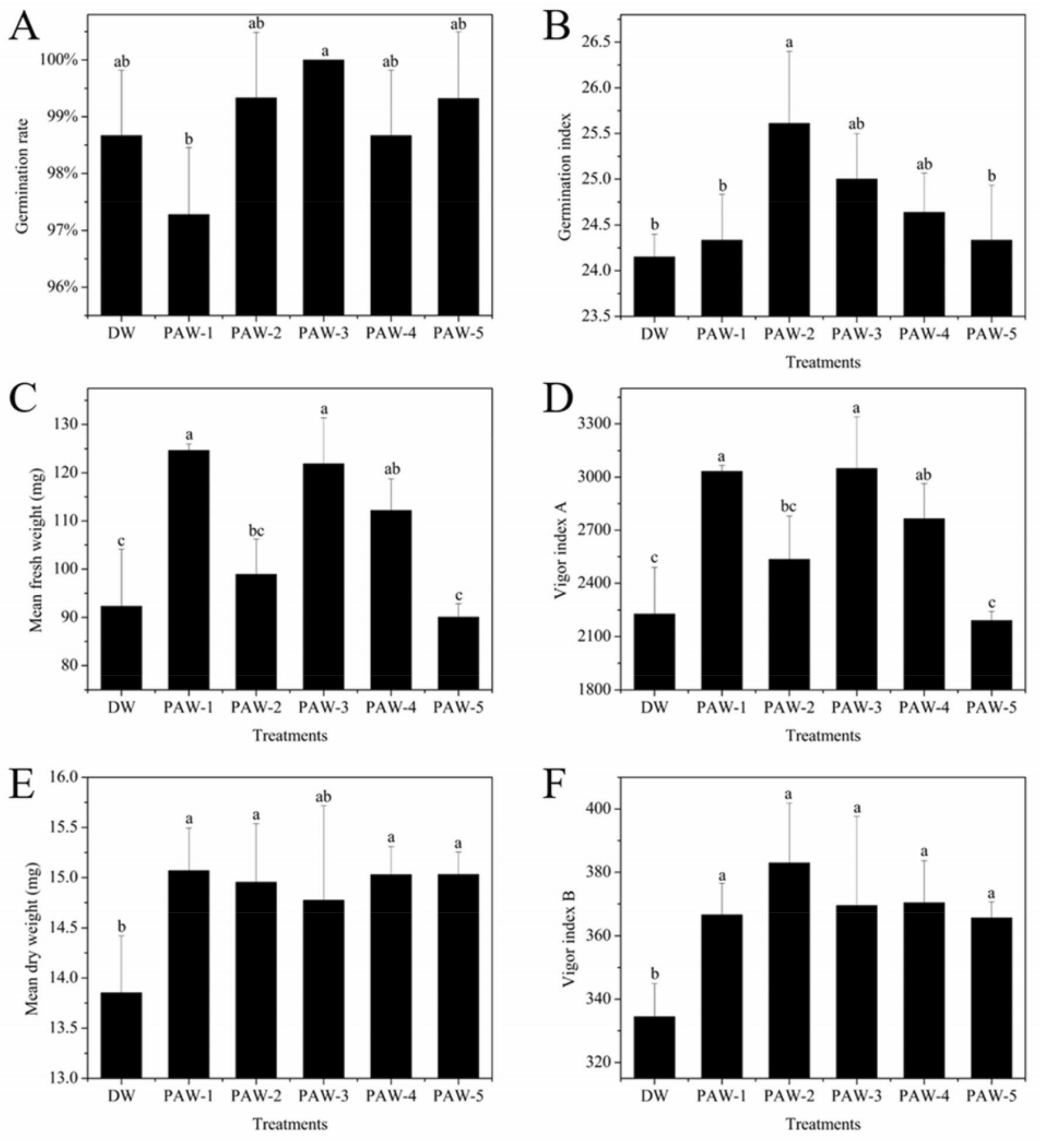
**A** Germination rate, **B** germination index, **C** mean fresh weight, **D** vigour index A, **E** mean dry weight and **F** vigour index B of wheat seeds treated by DW or PAWs

To access the effects of PAWs on the germination and early growth of wheat, wheat seeds were cultivated with different PAW for 7 days with DW used for the control. The germination and growth parameters of wheat seeds irrigated by DW and PAWs are presented in Fig. 3. The results showed that the germination rates of all samples were higher than 97% and the seeds watered with PAW-3 showed significantly higher germination rates than those watered with DW. Particularly, seeds irrigated with PAW-3 exhibited a 100% germination rate. Besides, using PAWs to water wheat seeds could increase the germination index as compared with using DW, but only PAW-2 showed a significant difference. In addition, after growth for 7 days, the mean fresh weight and vigour index A of wheat seedlings watered with PAW-1, PAW-3 and PAW-4 were significantly higher than with DW and that of wheat seedlings watered with PAW-2 was higher with no significant difference. Similarly, wheat seedlings watered with PAWs accumulated more dry matter and showed enhanced vigour index B. More specifically, the mean dry weight of wheat seedlings was increased by 6.64%-8.74% in 7 days of growth with PAWs. These results indicated that the current PAWs were able to stimulate wheat seed germination and seedling growth.

### Quality Attributes of Wheat Plantlet Juice

To further access the effects of PAWs on wheat plantlet growth and nutrition accumulation, wheat plantlets were cultivated and prepared as juice after 14 days. The quality attributes of juice derived from wheat plantlets watered with DW or PAWs for 14 days are shown in Table 1. Compared with DW, PAW-3 significantly increased the TSS of the wheat plantlet juice by 13.82%, whilst other PAWs increased the TSS below 4%. The measured protein concentrations slightly decreased in PAW-1 wheat plantlet juice, PAW-2 wheat plantlet juice and PAW-5 wheat plantlet juice with no significant difference. In comparison, PAW-3 and PAW-4 significantly increased the soluble protein contents by 19.48% and 7.79%, respectively. However, none of the PAWs caused increases in vitamin C levels in wheat plantlet juice, and in fact, a significant decrease was observed in all wheat plantlets juice, especially PAW-5 wheat plantlet juice showed reduced vitamin C contents by as much as 19.19%. In contrast, all the samples treated by PAW contained more chlorophyll a, chlorophyll b and carotenoids. Amongst them, PAW-3 led to the largest increase in chlorophyll a, chlorophyll b and carotenoids by 89.46%, 112.46% and 91.58%, respectively, and PAW-5 resulted in the smallest increase by 8.94%, 31.96% and 6.35%, respectively in comparison with that of DW.

**Table 1:** Quality attributes of juice derived from wheat plantlets treated with DW or PAWs

Parameters	Control	PAW-1	PAW-2	PAW-3	PAW-4	PAW-5
pH	6.04 ± 0.01c	6.07 ± 0.01b	6.07 ± 0.01b	6.07 ± 0.00b	6.09 ± 0.01a	6.07 ± 0.01b
TSS (Brix°)	3.69 ± 0.05d	3.79 ± 0.05bc	3.83 ± 0.00b	4.20 ± 0.05a	3.71 ± 0.05d	3.70 ± 0.05d
Soluble protein (g/100 g FW)	0.77 ± 0.02bc	0.71 ± 0.06c	0.70 ± 0.07c	0.92 ± 0.11a	0.83 ± 0.05ab	0.75 ± 0.04bc
Vitamin C (mg/100 g FW)	19.33 ± 0.94a	17.02 ± 1.20b	17.44 ± 0.85b	15.83 ± 0.55b	16.74 ± 0.63b	15.62 ± 1.49b
Chlorophyll a (μg/100 g FW)	401.80 ± 25.52d	607.33 ± 11.53b	532.69 ± 48.50c	761.25 ± 45.75a	564.12 ± 32.09bc	437.71 ± 18.65d
Chlorophyll b (μg/100 g FW)	106.54 ± 7.37e	181.57 ± 2.68bc	154.40 ± 12.97 cd	226.36 ± 12.94a	187.04 ± 29.51b	140.59 ± 15.69d
Carotenoids (μg/100 g FW)	58.30 ± 4.24c	88.54 ± 2.02b	79.81 ± 8.55b	111.69 ± 8.74a	78.21 ± 7.15b	62.00 ± 4.09c
TPC (mg GAE/100 g FW)	92.36 ± 2.40b	92.82 ± 2.18b	91.38 ± 3.76b	102.02 ± 5.08a	94.85 ± 2.70b	92.36 ± 2.89b
DPPH (μmol TE/g FW)	1.27 ± 0.04c	1.34 ± 0.02bc	1.37 ± 0.06b	1.46 ± 0.05a	1.38 ± 0.01b	1.47 ± 0.04a
ORAC (μmol TE/g FW)	12.93 ± 0.38c	14.29 ± 0.25bc	14.63 ± 0.87b	16.20 ± 0.73a	14.13 ± 0.36bc	17.50 ± 1.24a
POD (U/g FW)	84.37 ± 0.61b	105.18 ± 8.53a	67.12 ± 1.64c	79.23 ± 2.33b	63.40 ± 3.93c	68.63 ± 1.30c
PPO (U/g FW)	4.82 ± 0.00b	4.69 ± 0.47b	6.97 ± 0.00a	6.88 ± 0.25a	3.30 ± 0.25c	6.22 ± 0.49a
SOD (U/g FW)	33.57 ± 0.11d	42.96 ± 1.69c	45.01 ± 0.31bc	49.39 ± 0.72a	44.15 ± 0.31bc	47.01 ± 2.57ab
L*	58.36 ± 2.25bc	61.57 ± 0.10a	59.44 ± 0.38b	53.13 ± 0.16d	57.17 ± 0.09c	58.29 ± 0.06bc
a*	− 19.37 ± 0.06f	− 17.13 ± 0.05c	− 18.20 ± 0.09d	− 18.41 ± 0.02e	− 16.80 ± 0.03b	− 16.35 ± 0.02a
b*	51.31 ± 2.55a	53.10 ± 0.14a	52.88 ± 0.31a	48.33 ± 0.24b	51.09 ± 0.19a	52.66 ± 0.11a
∆E*	0	9.26	2.5	18.62	4.03	5.47

Means in the same row with different letters indicate significant difference (*n* = 3, *p* < 0.05)

The TPC was significantly increased only in PAW-3 wheat plantlet juice by 10.46% whilst other PAW wheat plantlet juice showed no significant enhancement when compared with the control. The changes in antioxidant capacity of wheat plantlet juice by PAW treatments were evaluated by DPPH and ORAC assay. In the case of the DPPH assay, PAWs (PAW-1 to PAW-5) resulted in different increases in antioxidant activity of wheat plantlet juice by 5.51%, 7.87%, 14.96%, 8.66% and 15.75%, respectively, whilst PAW-1 exhibited no significant difference from DW. With regard to the ORAC assay, ORAC values of PAW wheat plantlet juice (PAW-1 to PAW-5) were increased by 10.52%, 13.15%, 25.29%, 9.28% and 35.34%, respectively, but PAW-1 wheat plantlet juice and PAW-4 wheat plantlet juice showed no significant difference from DW wheat plantlet juice. As for enzyme activity, a 24.66% enhancement in POD activity of PAW-1 wheat plantlet juice was observed, whereas other PAWs decreased the POD activity of wheat plantlet juice at different degrees. In the case of PPO activity, treatments with PAW-2, PAW-3 and PAW-5 exhibited enhancement effects, however, PAW-1 and PAW-4 showed a debilitating effect. The greatest enhancement of 44.60% was achieved with PAW-2 treatment as compared with the control. In addition, PAWs (PAW-1 to PAW-5) resulted in 27.97%, 34.08%, 47.12%, 31.52% and 40.04% increase, respectively, in SOD activity compared with the control. Moreover, the most notable colour change was found in PAW-3 wheat plantlet juice, which became darker, less yellow and less green, as indicated by the changes in L*, a*, b* and ∆E* values, and PAW-1 was able to get the juice colour lighter and showed an obvious colour difference. In contrast, PAW-2, PAW-4 and PAW-5 exhibited less ability to change the colour of wheat plantlet juice.

### Free Amino Acids Profile

The contents of the free amino acids in wheat plantlet juice derived from DW or PAW treating plantlets are shown in Table 2. PAW treatments could significantly increase the total amount of free amino acids in the juice, and nearly all of the free amino acids increased in PAW wheat plantlet juice except that Cys decreased. Specifically, PAWs (PAW-1 to PAW-5) increased the total amount of free amino acids by 19.28%, 9.12%, 28.23%, 18.86% and 15.50%, respectively, when compared with the control. Individually, the levels of Thr, Val, Met, Leu, Phe, Trp, Ser, Ala, Tyr, Arg and γ-aminobutyric acid (GABA) were significantly increased in all PAW wheat plantlet juice, whilst Gly and Pro contents of PAW-1 wheat plantlet juice, Ile, Lys, His, Glu, Gly and Pro contents of PAW-2 wheat plantlet juice, Pro content of PAW-3 wheat plantlet juice, Gly content of PAW-4 wheat plantlet juice and His, Asp, Gly contents of PAW-5 wheat plantlet juice showed no significant difference from DW wheat plantlet juice. In particular, amongst 19 determined free amino acids, 18 free amino acids were significantly increased and 14 free amino acids levels were the highest in PAW-3 wheat plantlet juice, which suggested that PAW-3 treatment was the best for the accumulation of free amino acids in wheat seedlings.

**Table 2:** Amino acids (mg/100 g FW) profile of juice derived from wheat plantlets treated with DW or PAWs

Amino Acids	DW	PAW-1	PAW-2	PAW-3	PAW-4	PAW-5
Ala	8.77 ± 0.16e	11.01 ± 0.23c	10.51 ± 0.14d	12.13 ± 0.11b	12.46 ± 0.05ab	12.52 ± 0.04a
Arg	9.54 ± 0.16e	11.83 ± 0.14b	10.59 ± 0.18d	12.71 ± 0.01a	11.77 ± 0.20b	11.13 ± 0.06c
Asp	20.60 ± 0.10d	24.52 ± 0.37b	21.32 ± 0.39c	25.26 ± 0.20a	21.49 ± 0.24c	20.61 ± 0.05d
Cys	0.14 ± 0.01a	0.11 ± 0.00a	0.11 ± 0.01a	0.12 ± 0.01a	0.13 ± 0.02a	0.12 ± 0.02a
GABA	20.64 ± 0.09e	24.51 ± 0.22c	23.34 ± 0.39d	27.36 ± 0.20a	25.71 ± 0.19b	26.05 ± 0.05b
Glu	0.43 ± 0.01b	1.26 ± 0.23a	0.66 ± 0.13b	1.01 ± 0.03a	1.06 ± 0.00a	1.23 ± 0.00a
Gly	1.34 ± 0.15b	1.49 ± 0.17ab	1.43 ± 0.05b	1.73 ± 0.01a	1.54 ± 0.01ab	1.50 ± 0.05ab
His	20.36 ± 0.10d	22.52 ± 0.41b	20.10 ± 0.17d	24.09 ± 0.42a	21.74 ± 0.36c	20.82 ± 0.27d
Ile	9.86 ± 0.05d	11.01 ± 0.11b	10.08 ± 0.34d	11.89 ± 0.12a	11.00 ± 0.07b	10.49 ± 0.00c
Leu	6.18 ± 0.05d	7.26 ± 0.05b	6.89 ± 0.23c	8.00 ± 0.18a	7.51 ± 0.06b	7.22 ± 0.01b
Lys	15.77 ± 0.03c	18.27 ± 0.52b	16.48 ± 0.31c	19.78 ± 0.44a	18.71 ± 0.38b	17.89 ± 0.17b
Met	0.07 ± 0.02e	0.13 ± 0.03d	0.19 ± 0.00c	0.17 ± 0.02c	0.25 ± 0.01b	0.30 ± 0.00a
Phe	2.78 ± 0.05d	3.45 ± 0.05c	3.40 ± 0.19c	4.07 ± 0.01a	3.77 ± 0.02b	3.39 ± 0.01c
Pro	0.83 ± 0.03b	1.11 ± 0.13ab	1.10 ± 0.03ab	0.96 ± 0.08b	1.39 ± 0.09a	1.41 ± 0.32a
Ser	15.26 ± 0.02e	18.28 ± 0.31b	17.06 ± 0.30d	19.60 ± 0.08a	18.16 ± 0.11bc	17.79 ± 0.03c
Thr	8.49 ± 0.03e	10.37 ± 0.18b	9.53 ± 0.19d	11.25 ± 0.02a	10.24 ± 0.05b	9.90 ± 0.00c
Trp	2.48 ± 0.51b	3.58 ± 0.24a	3.32 ± 0.12a	3.55 ± 0.09a	3.36 ± 0.04a	3.31 ± 0.08a
Tyr	2.68 ± 0.00d	3.58 ± 0.03b	3.20 ± 0.09c	3.83 ± 0.12a	3.50 ± 0.04b	3.16 ± 0.05c
Val	13.94 ± 0.38e	16.73 ± 0.10b	15.44 ± 0.06d	17.83 ± 0.10a	16.54 ± 0.08bc	16.14 ± 0.04c
Total	160.14 ± 0.54e	191.02 ± 2.78b	174.74 ± 2.86d	205.35 ± 1.99a	190.34 ± 1.82b	184.96 ± 0.46c

Means in the same row with different letters indicate significant difference (*n* = 3, *p* < 0.05)

### Mineral Compositions

The results of elemental analysis of the juice derived from wheat plantlets treated by DW or PAWs are shown in Table 3. The data showed that Ca, P, K, S, Mg and Mn contents of all PAW wheat plantlet juice, Na content of PAW-2 wheat plantlet juice, PAW-3 wheat plantlet juice and PAW-4 plantlet juice, Fe content of PAW-3 wheat plantlet juice and Zn content of PAW-1 wheat plantlet juice, PAW-2 wheat plantlet juice, PAW-3 wheat plantlet juice and PAW-5 wheat plantlet juice were significantly increased when compared with that of DW wheat plantlet juice. Remarkably, applying PAW-3 significantly promoted the accumulation of all the mineral elements in wheat seedlings and PAW-3 wheat plantlet juice contained the highest content of all kinds of determined minerals amongst the samples. Specifically, the levels of Ca, P, Na, K, S, Mg, Mn, Fe, Zn of PAW-3 wheat plantlet juice were increased by 17.19%, 24.98%, 4.43%, 19.51%, 22.43%, 17.68%, 22.15%, 31.14% and 20.54%, respectively. However, Na content of PAW-5 wheat plantlet juice was significantly decreased. The results suggested that PAW treatments could enhance the accumulation of macroelements (Ca, P, Na, K, S and Mg) and microelements (Mn, Fe and Zn) during wheat seedling growth, and PAW-3 was the most suitable treatment.

**Table 3:** Minerals (μg/g FW) contents of juice derived from wheat plantlets treated with DW or PAWs

Minerals	DW	PAW-1	PAW-2	PAW-3	PAW-4	PAW-5
Ca	93.00 ± 0.90f	97.90 ± 0.35e	99.68 ± 0.40d	108.99 ± 0.31a	103.74 ± 0.30b	101.46 ± 1.32c
Fe	1.67 ± 0.03bcd	1.76 ± 0.10b	1.61 ± 0.05 cd	2.19 ± 0.04a	1.55 ± 0.02d	1.70 ± 0.02bc
K	984.44 ± 2.13d	1069.78 ± 5.28c	1116.44 ± 2.93b	1176.54 ± 8.92a	1068.02 ± 4.27c	1111.41 ± 11.17b
Mg	140.04 ± 1.36d	148.16 ± 0.49c	152.14 ± 0.76b	164.80 ± 0.09a	149.44 ± 0.37c	147.59 ± 2.28c
Mn	1.49 ± 0.02e	1.57 ± 0.00d	1.58 ± 0.02d	1.82 ± 0.02a	1.62 ± 0.02c	1.69 ± 0.00b
Na	21.66 ± 0.13d	22.02 ± 0.27 cd	22.43 ± 0.23ab	22.62 ± 0.20a	22.10 ± 0.13bc	19.64 ± 0.21e
P	620.57 ± 4.16e	667.67 ± 2.78d	682.97 ± 3.47c	775.60 ± 2.62a	725.83 ± 2.04b	725.33 ± 0.98b
S	244.50 ± 1.57e	257.52 ± 1.20d	255.97 ± 2.01d	299.34 ± 0.91a	268.68 ± 0.83b	266.22 ± 0.79c
Zn	1.12 ± 0.00d	1.19 ± 0.02b	1.14 ± 0.02bc	1.35 ± 0.07a	1.10 ± 0.02d	1.14 ± 0.00bc

Means in the same row with different letters indicate significant difference (*n* = 3, *p* < 0.05)

### Pearson Correlation Analysis of PAW Characteristics and Effects in Wheat

Pearson’s correlations between physicochemical properties of PAWs and wheat germination parameters, quality attributes of wheat plantlet juice, free amino acids and minerals were evaluated, respectively. As shown in Table S1, Pearson correlation coefficients in mean dry weight and vigour index B in association with pH were -0.860 and -0.801, those between the above germination parameters and EC were 0.659 and 0.543. With regard to NO_2_^−^ and NO_3_^−^, Pearson correlation coefficients were 0.681, 0.564 and 0.684, 0.561, respectively. However, there were nearly no correlations between mean fresh weight, vigour index A and EC, NO_2_^−^ and NO_3_^−^, and other germination parameters showed weak correlations with PAW characteristics. The extremely significant positive correlations were obtained amongst EC, NO_2_^−^ and NO_3_^−^, and the extremely significant negative correlations were obtained between pH and EC, NO_2_^−^ and NO_3_^−^, respectively. In general, the promoting effect of PAWs on wheat seed germination and seedling growth depended on NO_2_^−^ and NO_3_^−^.

The results of Pearson correlation analysis of PAW characteristics and quality attributes, free amino acids and minerals of wheat plantlet juice were shown in Tables S2, S3 and S4, respectively. The data showed that there were negative correlations between pH and most of the determined indexes except for vitamin C, POD, Cys and Na, whilst the opposite results were obtained of the correlations between other determined PAW compositions and indexes of wheat plantlet juice. However, Pearson correlation coefficients of NO_2_^−^ and NO_3_^−^ in association with TSS, carotenoids, PPO, Asp, Cys, Fe and Zn were below 0.2. Remarkably, NO_2_^−^ or NO_3_^−^ exhibited significant positive correlations with DPPH, Met, Ala, Pro, GABA and P. In summary, the enhancements of nutritional materials of wheat plantlet juice and antioxidant activities could be attributed to the nitrite and nitrate of PAWs.

## Discussion

With the growing population and the outbreak of COVID-19 throughout the world, food shortages are becoming more and more serious (FAO et al. 2020), which challenges people to find more efficient and sustainable crop production methods to address the basic food need. In fact, researchers have tried a variety of novel physical treatment techniques including ultrasound, pulsed electric field, irradiation, magnetic field and cold plasma, to solve this worldwide problem and cold plasma is the most impressive amongst these techniques (Ahmed et al. 2020; AlSalhi et al. 2019; Balakhnina et al. 2015; Guimarães et al. 2020; Ndiffo Yemeli et al. 2020). In the meantime, wheat (*Triticum aestivum* L.) is consumed in huge quantities as one of the most important crop plants throughout the world. In this case, the current study chose wheat to demonstrate the effects of indirect cold plasma (PAW) treatments on its germination, early growth and nutrition accumulation, so as to provide an effective method to improve crop production.

### Generation of PAWs

Numerous studies clarified that the characteristics of PAW depended on different plasma parameters such as types of discharge, discharge pressure, discharge distance, electrode configuration, power supply, types of the feed gas, gas flow, treatment time, and water volume (Kim and Kim 2021; Thirumdas et al. 2018; Zhou et al. 2020). In our study, argon and oxygen reactive species were generated by an atmosphere pressure Ar–O_2_ plasma discharge to activate the water and nitrate and nitrite were formed in PAWs, which was in line with those reported previously (Ali et al. 2021; Chizoba Ekezie et al. 2018). However, other studies (Karmakar et al. 2021; Vlad and Anghel 2017) found that there were weak emission lines of OH radicals and nitrogen reactive species in open atmosphere Ar or Ar–O_2_ cold plasma, which was not shown in the current research. The reason might be that argon and oxygen active species generated in plasma would motivate the emission of ambient air and the distance between the OES probe and plasma plume, which was just 5 mm in the current research, affected the results. In addition, at the same distance, a slight change in the orientation of the OES probe would also influence the result. Vlad and Anghel (2017) reported that when treatment time varied from 10 to 50 min, argon-discharged PAW contained a higher concentration of H_2_O_2_, whilst air-discharged PAW contained a higher concentration of RNS. Whereas, in the current study, H_2_O_2_ could not be detected in all PAWs, which was reasonable as a higher water volume of 75 mL was used in the current study than most other studies, and only 3.34 ppm NO_3_^−^ was thus generated for treatment for 5 min. As a result, the effects of NO_2_^−^ and NO_3_^−^ rather than the combined effects of NO_2_^−^, NO_3_^−^ and H_2_O_2_ on wheat seeds could be analysed in the current study. In the meantime, our results showed that the formation of NO_2_^−^ and NO_3_^−^ resulted in reduced pH and increased EC, which was consistent with other studies. For example, Sergeichev et al. (2021) investigated the physicochemical properties of pure water treated by an Ar microwave plasma jet and showed that EC values and NO_2_^−^ and NO_3_^−^ concentrations increased linearly with plasma exposure times. Similarly, Zhou et al. (2019) showed the same change tendency with lower pH and higher RONS in PAW treated with N_2_, He, Air and O_2_ plasma. Moreover, the formation process of RNS in PAW was well summarized (Bradu et al. 2020) and not repeated in the current study.

### Effects of PAWs on Wheat Seed Germination

It is widely acknowledged that the biological activity of PAW was attributed to NO_2_^−^, NO_3_^−^ and H_2_O_2_. Previous studies showed that H_2_O_2_ could diffuse through the plant cell membrane and act as a signalling molecule mediating in the early growth during imbibition and germination (Ranieri et al. 2020), yet the positive effects of PAW in the current study could not be associated with it. In PAWs, NO_3_^−^ and NO_2_^−^ could serve as the source of N and be metabolized by wheat seed, which was the key effector, on the other hand, they could be reduced to NO, which modulated various hormones controlling seed dormancy and germination such as abscisic acid, gibberellins and indoleacetic acid (Chen et al. 2020; Mu et al. 2018). And the content of plant nitrogen was closely related to photosynthesis and further affected the accumulation of dry matter in crops (Verkroost and Wassen 2005), which explained why wheat seeds exhibited higher mean dry weight and seed vigour when irrigated with PAWs as reported in the current study. However, there was a limited range of beneficial RNS concentrations, and concentrations too high or too low were detrimental to crop growth. For example, Fan et al. (2020) investigated the effect of PAW on mung beans and showed that NO_3_^−^ concentrations higher than 20 mg/mL could not promote the growth of stem and root, and for NO_3_^−^ concentrations reaching 118.39 mg/mL, the germination rate decreased. In contrast, Lo Porto et al. (2018) found that PAW containing 1.24 or 10.54 mg/L of NO_3_^−^ exhibited a positive effect on soybean seed germination and growth. Similarly, in the current study, the total concentrations of NO_3_^−^ and NO_2_^−^ in PAWs were below 5 mg/L and all the PAWs exhibited enhanced effects on wheat seed germination and growth at different degrees, especially for PAW-3, the best overall improvement was achieved. Notably, pH < 4.5 and EC > 3.0 dS/m were not recommended for crop growth because they would affect enzyme activity and water absorption of seeds (Ranieri et al. 2020). Research on the effects of pH on the uptake of 0.5 mM NO_2_^−^ and NO_3_^−^ of 7-day-old wheat seedlings demonstrated that the nitrate uptake was not affected by pH, and the nitrite uptake at pH 4 hardly increased with time, whilst its amount was comparable to nitrate at pH 6.5 suggesting that NO_2_^−^ at low pH was strong oxidant and toxic substance as HNO_2_ (Zsoldos et al. 1999). A similar report showed that the dry matter of shoot of the common wheat seedlings was increased linearly with 0–0.5 mM nitrite at pH 4, whilst NO_2_^−^ content beyond 0.5 mM could cause stress for the root growth (Bona et al. 2001). Amongst the PAWs generated in our study, the nitrite concentrations of all PAWs were below 0.1 mM, but only PAW-1 achieve the recommended pH value, suggesting that abiotic stress might occur in long-term cultivation. Whereas PAWs showed limited enhancement on germination rate because the germination rate of all samples was higher than 97%, which is the normal rate for fresh seeds (Tian et al. 2019). Moreover, extremely weak correlations between germination index and NO_2_^−^ and NO_3_^−^ were obtained in our study which suggested that germination velocity did not depend on the RNS of PAWs. Similarly, Kučerová et al. (2019) quantified the NO_2_^−^, NO_3_^−^ and H_2_O_2_ of plasma-activated tap water with and without the presence of wheat seeds for 7 days. Without seeds, the concentration of H_2_O_2_ decreased slowly and the residue could still be determined after 6 days. In the meantime, the concentrations of NO_2_^−^ and NO_3_^−^ remained stable. With the presence of seeds, the concentration of H_2_O_2_ decreased rapidly in several minutes and could not be detected after 18 h, whilst the concentration of NO_2_^−^ and NO_3_^−^ started to decrease after 24 h when seeds started to germinate. The authors, therefore, concluded that seeds interacted with H_2_O_2_ mainly in the early stage of imbibition and germination, whereas NO_2_^−^ and NO_3_^−^ were metabolized after germination. Another explanation was that the transport of NO_3_^−^ was relatively slower than that of H_2_O_2_ as prior was regulated by specialized membrane transport proteins (Forde 2000). Mean fresh weight was the reflection of water absorption during wheat growth and Pearson correlation coefficients showed that NO_2_^−^ and NO_3_^−^ in PAWs were not the cause of the enhancements in wheat seedling fresh weight. Scanning electron micrograph images of mung bean seed treated by PAW showed that the seed coat was eroded and chapped which resulted in the enhancement in water absorption (Zhou et al. 2019). Whereas the salinity of PAWs caused the increase in osmotic pressure and high osmotic pressure would decrease the water uptake of the seeds by regulating the transcellular transport path (Knipfer et al. 2021; Yue et al. 2018). As shown in Fig. 3, taken as a whole, the mean fresh weight of wheat seeds treated by PAWs showed first increasing and then decreasing tendency, but only PAW-5 did not increase it as compared with the control, which might result from the combined effect of PAWs on the improvement of seed coat wettability and increasing of osmotic potential. Similar results were obtained in wheat with 0.5 min/mL to 3 min/mL PAW (Kučerová et al. 2019). As germination index and mean dry weight of it showed a significant difference from the control, it was supposed that water loss during sampling of wheat seeds treated by PAW-2 resulted in no significant increase in mean fresh weight. Nevertheless, it did not affect our conclusion that PAWs exhibited promotion effects on wheat seed germination and seedling growth for 7 days.

### Effects of PAWs on Quality Attributes of Wheat Plantlets Juice

Wheat plantlets grown for 8–15 days are normally acknowledged for high nutritional value and are commonly consumed as raw juice (Bianchi et al. 2019). In the current study, PAWs especially PAW-3 could greatly improve the nutritional value of juice derived from wheat plantlet grown for 14 days by increasing TSS, protein content, pigments, TPC, free amino acids and mineral elements, which was consistent with the performance of PAWs in the germination test. Nutrient status reflected the condition of plant growth and development. Carbohydrates were strongly required in dividing and differentiating cells and their metabolism was important for plant growth and previous study selected soluble sugar content as the evaluation index for wheat seedling growth (Eveland and Jackson 2012; Zhu et al. 2021). Although PAW-3 increased 13.82% of TSS, the correlation coefficients indicated that it was not related to the determined parameters of PAW. A previous report found that 0.1 mM NO_3_^−^ treatment could significantly increase the plant dry weight, leaf soluble protein and plant N content of wheat plantlet grown for 24–37 days and result in a significant positive correlation between plant N content and plant dry weight, but plant N content and leaf soluble protein content were not significantly correlated which could be attributed to the root to shoot dry weight ratio (Andrews et al. 1999). A similar result was obtained in the current study that NO_2_^−^ and NO_3_^−^ of PAWs showed weak correlations with the soluble protein content of wheat plantlet juice. In addition, another study demonstrated that applying nitrate-nitrogen increased the NO_3_^−^ and reduced the vitamin C content of spinach cultivated for 9 days, and the transfer of the spinach to N-free media would recover the vitamin C and reduce the NO_3_^−^ level as vitamin C was recognized as the inhibiter of N-nitroso compounds formed from nitrite (Mozafar 1996). In the same way, NO_2_^−^ and NO_3_^−^ of PAWs coexisted and significantly decreased the vitamin C content of wheat plantlet juice. It is reported that RNS stimulated the formation of pigments by upregulating the amounts of stromal and thylakoid proteins of leaves and increasing the chloroplasts during leaf growth (Than et al. 2021). Chlorophyll a and chlorophyll b are essential in photosynthesis influencing the synthesis of organic matter in land plants and their contents reflect the plant nitrogen status (Tanaka and Tanaka 2011). Our results showed that PAWs could improve the utilization of nitrogen in wheat plantlets and PAW-3 performed best, indicating that the levels of NO_2_^−^ and NO_3_^−^ did not dominate the nitrogen transformation which was in line with the Pearson correlation coefficients. In addition, chlorophyll was found to increase with increasing stress levels in stress-tolerant plants which suggested that it might be a potential indicator of stress resistance (Wang et al. 2022). Thus, the downward trend of chlorophyll from PAW-3 wheat plantlet juice to PAW-5 plantlet juice was supposed to result from the abiotic stress caused by low pH and increasing NO_2_^−^ content after 14 days of growth. Carotenoids and TPC are widely acknowledged for their biological functions such as antioxidation, anticancer action and immunological enhancement (Maoka 2020). The increased carotenoids, TPC and antioxidant activities of PAW wheat plantlet juice suggested that PAW treatment could improve the physiological functions of wheat plantlet juice. PPO and POD belong to spoilage enzymes that can result in the browning of juices, and the inactivation of these two enzymes has positive effects on browning reduction (Jimenez-Sanchez et al. 2017). However, in the case of raw juice, the retention of PPO and POD activities are beneficial for human health (Skoczylas et al. 2018). In this aspect, PAW-1 and PAW-3 showed advantages in the preservation of POD and PPO activities. Moreover, POD and SOD were enzyme antioxidant systems activated for ROS scavenging and metabolism to alleviate oxidative damage. Tari and Csiszár (2003) found that 1 mM nitrite at pH = 4 led to nitrosative stress and reduction of POD activity in wheat. Kučerová et al. (2021) compared the effects of PAW, H_2_O_2_ and NO_3_^−^ on Lettuce growth and found that only 0.85 mM NO_3_^−^ increased the SOD activity. In the current study, SOD activity of PAW wheat plantlet juice was increased which could be related to NO_3_^−^ in PAWs. However, only POD activity of PAW-1 wheat plantlet juice was increased suggesting that PAW-2, PAW-3, PAW-4 and PAW-5 might cause the nitrosative stress in wheat plantlets cultivated for 14 days.

### Effects of PAWs on Free Amino Acids Profile

Nitrogen is utilized directly for synthesizing amino acids, with an initial process involving the synthesis of glutamine and glutamate via the 2-oxoglutarate-glutamate synthase pathway (Rossi et al. 2021), and the enhancement of free amino acids could thus be attributed to the NO_2_^−^ and NO_3_^−^ occurring in PAWs. GABA and proline are able to improve abiotic stress tolerance in various plants through free radical-scavenging, osmotic adjustment and chlorophyll metabolism (Abd El-Gawad et al. 2021; Li et al. 2016; Rossi et al. 2020). Al-Quraan et al. (2013) reported that salt stress (100 mM NaCl) and osmotic stress (200 mM sorbitol or 200 mM mannitol) could result in a drastic increase of GABA and chrolophy b metabolites in wheat. Guo et al. (2018) found that drought stress induced by water deficiency would increase the proline metabolites and decrease the photosynthetic pigment metabolites of wheat plantlets grown for 4 weeks. On this basis, the increase of GABA and proline contents of PAW wheat plantlet juice in the current study was supposed to be associated with the osmotic stress and oxidative stress caused by low pH, NO_2_^−^ and NO_3_^−^ of PAWs, and the variation of enhancements in GABA and proline amongst PAWs might result from the different levels of stresses. Moreover, plant responses to combined stresses were complex and involved many changes in gene expression and metabolites, which was different from the responses to individual stress (Carillo 2018). For example, it was found that GABA accumulation under salinity in durum wheat plants only appeared with high nitrate and high light, and in these conditions, plants exhibited lower ROS levels and higher photosynthetic efficiency than plants under salinity at low light (Woodrow et al. 2017). As increased free amino acid in PAW wheat plantlets, therefore, wheat plantlets irrigated by PAWs might exhibit higher abiotic stress tolerance and alleviate the deleterious effects caused by worse climate and environment.

### Effects of PAWs on Mineral Compositions

Mineral composition analysis indicated the variations of mineral concentrations in wheat plantlets as affected by PAW treatments. Macroelements (Ca, P, Na, K, S and Mg) and microelements (Mn, Fe and Zn) are essential for plant growth and human health (Kopriva 2015; Martínez-Ballesta et al. 2011). Therefore, the application of PAWs especially PAW-3 could enhance wheat plantlet growth by promoting minerals absorption. Albornoz and Lieth (2015) found that nitrate absorption had a positive correlation with the absorption of H_2_PO_4_^−^, K^+^, Ca^2+^, Mg^2+^ and SO_4_^2−^ for lettuce roots. Li et al. (2007) investigated the influence of nitrate on metal sorption and bioaccumulation in marine phytoplankton and clarified that 6–55 μmol/L of nitrate greatly affected the absolute adsorption of Fe, Zn and Mn. However, Pearson correlation analysis showed that the enhancements of Na, Fe and Zn contents were not related to the levels of NO_2_^−^ and NO_3_^−^ of PAWs. Furthermore, an acidic condition created by NO_2_^−^ and NO_3_^−^ in PAWs would reduce the absorption of minerals. As zinc was a constituent of enzyme carbonic anhydrase, alcoholic dehydrogenase and superoxide dismutase and iron was a component of porphyrin compounds, it was speculated that abiotic stresses induced by low pH, NO_2_^−^ and NO_3_^−^ affected the absorption of Zn and Fe. In agreement with our proposal, (Chen et al. 2011) reported that the levels of Fe and Zn of wheat seedlings were decreased under salt stress and supplementation with antioxidant (ascorbic acid or *N*-acetyl-l-cysteine) increased the accumulation of Fe and Zn. Moreover, Xu et al. (2014) evaluated the effect of Zn on salt tolerance of wheat and elucidated that sufficient Zn nutrition maintained antioxidant enzyme activities and reduced ROS over-accumulation in 20-day-old wheat seedlings. In the meantime, they also revealed that sufficient Zn nutrition decreased the Na^+^ accumulation by upregulating the expression of Na^+^/H^+^ antiporter genes, *TaSOS1* and *TaNHX1* to improve salt tolerance in wheat seedlings. Therefore, the enhancement of minerals absorption of wheat plantlets in the current study was due to the increased nitrogen sources and beneficial low abiotic stresses induced by pH, NO_2_^−^ and NO_3_^−^ of PAWs.

Altogether, it was concluded that plasma-activated water generated by atmosphere pressure Ar–O_2_ plasma jet for 1–5 min could improve wheat seed germination, seedling growth and nutrient accumulation of wheat plantlets which was attributed to the increased nitrogen supply and induced beneficial abiotic stresses of PAWs. Remarkably, PAW-3 was the best choice for the stimulation of wheat growth. However, the mechanism of PAW on wheat seed could not be fully explained by the current limited research and further research was needed to elucidate it.

## Conclusions

In the current study, it was demonstrated that PAWs generated by atmosphere pressure Ar–O_2_ plasma jet possessed the ability to enhance wheat seed germination, seedling growth and nutritional properties of wheat plantlet juice, which could be attributed to the increased nitrogen supply and induced beneficial abiotic stresses of PAWs. Results indicated that PAW-3 could not only accelerate the germination and growth of wheat seeds in 7 days but could also significantly enhance the nutrients in the juice extracted from wheat plantlets grown for 14 days such as TSS, protein content, pigments, TPC, free amino acids and mineral elements. Besides, PAW-3 exhibited the possibility to improve stress resistance of wheat plantlets by increasing the SOD activity, GABA and proline content. Moreover, PAW-3 showed the highest ability in changing the colour of wheat plantlet juice, although this influence could not be simply judged as beneficial or harmful. The current results suggested that PAWs could be a potential green technology for agriculture production.

## Supplementary Information

Below is the link to the electronic supplementary material.

Supplementary file1 (DOCX 25 kb)
