# Supplementary material for: Enhancement of Wheat Seed Germination, Seedling Growth and Nutritional Properties of Wheat Plantlet Juice by Plasma Activated Water
Source: J Plant Growth Regul. 2022 May 31;42(3):2006–22. doi: 10.1007/s00344-022-10677-3 (PMC9152647; doi:10.1007/s00344-022-10677-3)
Supplement: Supplementary file 1 — Supplementary file1 (DOCX 25 kb) [file 344_2022_10677_MOESM1_ESM.docx]

**Supplementary Materials**

Enhancement of Wheat Seed Germination, Seedling Growth and Nutritional Properties of Wheat Plantlet Juice by Plasma Activated Water

Junhong Wang^a, b, c^, Junhu Cheng^a, b, c^, Da-Wen Sun^a, b, c, d,^ ^[[1]](#footnote-1)^*

^a^ School of Food Science and Engineering, South China University of Technology, Guangzhou 510641, China

^b^ Academy of Contemporary Food Engineering, South China University of Technology, Guangzhou Higher Education Mega Center, Guangzhou 510006, China

^c^Engineering and Technological Research Centre of Guangdong Province on Intelligent Sensing and Process Control of Cold Chain Foods, & Guangdong Province Engineering Laboratory for Intelligent Cold Chain Logistics Equipment for Agricultural Products, Guangzhou Higher Education Mega Centre, Guangzhou 510006, China

^d^ Food Refrigeration and Computerized Food Technology (FRCFT), Agriculture and Food Science Centre, University College Dublin, National University of Ireland, Belfield, Dublin 4, Ireland

**Table S1.** Pearson correlation coefficients between PAW characteristics and germination parameters.

| Parameters | pH | EC | NO_2_^−^ | NO_3_^−^ |
| --- | --- | --- | --- | --- |
| Germination rate | -0.388 | 0.464 | 0.464 | 0.452 |
| Germination index | -0.390 | 0.158 | 0.170 | 0.159 |
| Mean fresh weight | -0.189 | -0.071 | -0.009 | -0.040 |
| Vigor index A | -0.244 | -0.041 | 0.021 | -0.011 |
| Mean dry weight | -0.860* | 0.659 | 0.681 | 0.684 |
| Vigor index B | -0.801 | 0.543 | 0.564 | 0.561 |

Note: *p < 0.05 and **p < 0.01.

**Table S2.** Pearson correlation coefficients between PAW characteristics and quality attributes of wheat plantlet juice.

| Parameters | pH | EC | NO_2_^−^ | NO_3_^−^ |
| --- | --- | --- | --- | --- |
| TSS | -0.237 | 0.054 | 0.109 | 0.077 |
| Soluble protein | -0.249 | 0.306 | 0.341 | 0.302 |
| Vitamin C | 0.914* | -0.837* | -0.867* | -0.863* |
| Chlorophyll a | -0.390 | 0.137 | 0.200 | 0.165 |
| Chlorophyll b | -0.578 | 0.355 | 0.414 | 0.381 |
| Carotenoids | -0.345 | 0.075 | 0.138 | 0.105 |
| TPC | -0.288 | 0.228 | 0.280 | 0.241 |
| DPPH | -0.878* | 0.851* | 0.873* | 0.869* |
| ORAC | -0.577 | 0.647 | 0.653 | 0.669 |
| POD | 0.539 | -0.676 | -0.644 | -0.643 |
| PPO | -0.205 | 0.094 | 0.106 | 0.113 |
| SOD | -0.911* | 0.749 | 0.784 | 0.775 |

Note: *p < 0.05 and **p < 0.01.

**Table S3.** Pearson correlation coefficients between PAW characteristics and free amino acids of wheat plantlet juice.

| Parameters | pH | EC | NO_2_^−^ | NO_3_^−^ |
| --- | --- | --- | --- | --- |
| Thr | -0.689 | 0.497 | 0.554 | 0.529 |
| Val | -0.716 | 0.531 | 0.586 | 0.563 |
| Met | -0.905* | 0.969** | 0.957** | 0.967** |
| Ile | -0.506 | 0.363 | 0.423 | 0.391 |
| Leu | -0.779 | 0.629 | 0.680 | 0.655 |
| Phe | -0.770 | 0.599 | 0.648 | 0.619 |
| Trp | -0.776 | 0.514 | 0.559 | 0.550 |
| Lys | -0.668 | 0.559 | 0.611 | 0.584 |
| His | -0.288 | 0.141 | 0.205 | 0.172 |
| Asp | -0.133 | -0.125 | -0.058 | -0.085 |
| Ser | -0.743 | 0.558 | 0.612 | 0.590 |
| Glu | -0.690 | 0.603 | 0.633 | 0.635 |
| Gly | -0.637 | 0.519 | 0.573 | 0.542 |
| Ala | -0.944** | 0.913** | 0.936** | 0.927** |
| Cys | 0.471 | -0.155 | -0.183 | -0.194 |
| Tyr | -0.629 | 0.391 | 0.450 | 0.423 |
| Arg | -0.671 | 0.491 | 0.547 | 0.521 |
| Pro | -0.787 | 0.840* | 0.824* | 0.838* |
| GABA | -0.872* | 0.784 | 0.824* | 0.808 |
| Total | -0.701 | 0.536 | 0.591 | 0.567 |

Note: *p < 0.05 and **p < 0.01.

**Table S4.** Pearson correlation coefficients between PAW characteristics and minerals of wheat plantlet juice.

| Parameters | pH | EC | NO_2_^−^ | NO_3_^−^ |
| --- | --- | --- | --- | --- |
| Ca | -0.793 | 0.696 | 0.737 | 0.710 |
| P | -0.845* | 0.788 | 0.823* | 0.801 |
| Na | 0.193 | -0.438 | -0.402 | -0.436 |
| K | -0.769 | 0.585 | 0.624 | 0.611 |
| S | -0.616 | 0.533 | 0.582 | 0.552 |
| Mg | -0.573 | 0.373 | 0.424 | 0.396 |
| Mn | -0.701 | 0.644 | 0.686 | 0.666 |
| Fe | -0.114 | 0.011 | 0.066 | 0.041 |
| Zn | -0.158 | -0.001 | 0.057 | 0.031 |

Note: *p < 0.05 and **p < 0.01.

1. * Corresponding author. Email: dawen.sun@ucd.ie, URLs: http://www.ucd.ie/refrig; http://www.ucd.ie/sun [↑](#footnote-ref-1)
